# Saliva Proteins of Vector *Culicoides* Modify Structure and Infectivity of Bluetongue Virus Particles

**DOI:** 10.1371/journal.pone.0017545

**Published:** 2011-03-14

**Authors:** Karin E. Darpel, Kathrin F. A. Langner, Manfred Nimtz, Simon J. Anthony, Joe Brownlie, Haru-Hisa Takamatsu, Philip S. Mellor, Peter P. C. Mertens

**Affiliations:** 1 Pirbright Laboratory, Vector-borne Disease Programme, Institute for Animal Health, Woking, United Kingdom; 2 Immunology Unit, University of Veterinary Medicine, Hannover, Germany; 3 Helmholtz Centre for Infection Research, Braunschweig, Germany; 4 Department of Pathology and Infectious Diseases, Royal Veterinary College, University of London, Hatfield, United Kingdom; Global Viral Forecasting Initiative, United States of America

## Abstract

Bluetongue virus (BTV) and epizootic haemorrhagic disease virus (EHDV) are related orbiviruses, transmitted between their ruminant hosts primarily by certain haematophagous midge vectors (*Culicoides* spp.). The larger of the BTV outer-capsid proteins, ‘VP2’, can be cleaved by proteases (including trypsin or chymotrypsin), forming infectious subviral particles (ISVP) which have enhanced infectivity for adult *Culicoides*, or KC cells (a cell-line derived from *C. sonorensis*). We demonstrate that VP2 present on purified virus particles from 3 different BTV strains can also be cleaved by treatment with saliva from adult *Culicoides*. The saliva proteins from *C. sonorensis* (a competent BTV vector), cleaved BTV-VP2 more efficiently than those from *C. nubeculosus* (a less competent / non-vector species). Electrophoresis and mass spectrometry identified a trypsin-like protease in *C. sonorensis* saliva, which was significantly reduced or absent from *C. nubeculosus* saliva. Incubating purified BTV-1 with *C. sonorensis* saliva proteins also increased their infectivity for KC cells ∼10 fold, while infectivity for BHK cells was reduced by 2–6 fold. Treatment of an ‘eastern’ strain of EHDV-2 with saliva proteins of either *C. sonorensis* or *C. nubeculosus* cleaved VP2, but a ‘western’ strain of EHDV-2 remained unmodified. These results indicate that temperature, strain of virus and protein composition of *Culicoides* saliva (particularly its protease content which is dependent upon vector species), can all play a significant role in the efficiency of VP2 cleavage, influencing virus infectivity. Saliva of several other arthropod species has previously been shown to increase transmission, infectivity and virulence of certain arboviruses, by modulating and/or suppressing the mammalian immune response. The findings presented here, however, demonstrate a novel mechanism by which proteases in *Culicoides* saliva can also directly modify the orbivirus particle structure, leading to increased infectivity specifically for *Culicoides* cells and, in turn, efficiency of transmission to the insect vector.

## Introduction

Arboviruses are transmitted between their mammalian hosts by arthropod vectors (ticks, insects), which also become productively infected by the virus. It has been demonstrated that in certain cases the saliva from these arthropods can play an important role in pathogen transmission mechanisms [1]. For example, West Nile virus [2], Sindbis virus [3], Cache valley virus [4] and vesicular stomatitis virus [5], [6] are all more infectious and/or more virulent if administered with saliva from their arthropod vector.

These effects are thought to be achieved primarily through the effect of insect or tick saliva components on the mammalian immune response [7]. Depending on saliva concentration, it was suggested that these effects could involve an immune-response modulation, which down-regulates specific antiviral cytokines [6], [8], [9]. Alternatively the saliva could cause immune-suppression in the mammalian host, enhancing virus infectivity and affecting the hosts' chances of survival [10], [11].

The *Bluetongue virus* (BTV) and *Epizootic haemorrhagic disease virus* (EHDV) species are both members of the genus *Orbivirus*, family *Reoviridae*. BTV is one of the most economically important arboviruses of domesticated ruminants (for review see [12]). Infected sheep and some species of deer frequently develop a severe haemorrhagic disease called bluetongue (BT), while a large proportion of infected cattle and other wild ruminants may only develop subclinical infections [13], [14]. Since 2006 Europe has experienced the worst ‘single’ BT outbreak ever recorded (caused by a ‘western’ strain of BTV-8 [15]), affecting many European countries, including those in the north of the region with no previous history of the disease (e.g. the Netherlands, Belgium, Germany, France and the UK) with case fatality rates up to 50% [16].

EHDV can also cause severe disease in both deer and cattle and has recently been included in the OIE notifiable disease list (http://www.oie.int/eng/maladies/en_classification2009).

The orbivirus genome is composed of ten segments of linear dsRNA, which code for a total of 10 distinct viral proteins: including three different non-structural proteins, and seven structural proteins. Proteins VP1, VP3, VP4, VP6 and VP7 form the icosahedral virus-core particle, which is surrounded by an outer capsid layer composed of VP2 and VP5 [17]. These outer-capsid components are the most variable of the viral proteins, and are primarily involved in cell attachment and penetration of the host cell during the early stages of infection [18], [19], [20]. They also represent a target for neutralising antibodies (particularly VP2) that are generated during infection of the mammalian host and therefore determine the serotype of the individual strain [21]. All of the virus genome segments, including those coding for the outer capsid proteins, also show sequence variations that correlate with the geographic origins of the virus isolate (topotype) [15], [22]. The most significant of these can be used to divide both BTV and EHDV isolates into two major ‘Eastern’ and ‘Western’ groups. Proteases (such as chymotrypsin) can cleave VP2, generating infectious subviral particles (ISVP) which have an enhanced infectivity for adult *Culicoides* and KC cells (a cell-line derived from *C. sonorensis*) [23].

BTV and EHDV are transmitted between their ruminant hosts almost exclusively by adult female *Culicoides* (one of the smallest of the haematophagous insects) [24]. Although *Culicoides* saliva is thought likely to play an important role in orbivirus transmission mechanisms, its effects on the transmission efficiency, infectivity or virulence of these viruses has not previously been reported. Our knowledge of *Culicoides* saliva proteins and their functions had been limited due to difficulties in collecting saliva from such small insects. With the development of more advanced molecular biology methods, cDNA expression libraries have been generated for the salivary gland and midgut of *C. sonorensis* [25], as well as the salivary gland of *C. nubeculosus* [26]. These techniques have the potential to provide greater access to individual (expressed) protein components of the insect's saliva. However, they will not provide information on their relative abundance in saliva, or the effects of *Culicoides* saliva (as a whole) on virus structure and transmission.

We previously described an efficient method for the collection of relatively large quantities of *Culicoides* saliva in a pure form, using protein binding filters [27]. Indeed, sufficient saliva proteins were collected from *C. nubeculosus* (an inefficient BTV vector from Europe) for direct protein sequencing by mass spectrometry [27]. Here we report the effect of saliva proteins from a competent BTV / EHDV vector (*C. sonorensis*) and an inefficient vector (*C. nubeculosus*) on the structure (and outer capsid components) of purified BTV and EHDV particles, as well as their effect on the infectivity of BTV for KC cells (derived from *C. sonorensis*). The results obtained show that proteases in the saliva of adult *Culicoides* can directly modify the structure and the infectivity of the orbiviruses they transmit, through cleavage of the outermost viral protein. The efficiency of cleavage is shown to be dependent on temperature, protein composition of the saliva (which varies between different *Culicoides* spp.) and the protease susceptibility of VP2 from individual virus strains.

## Results

### Identification of a ∼29 kD trypsin protein in *C. sonorensis* saliva

**Figure 1:**
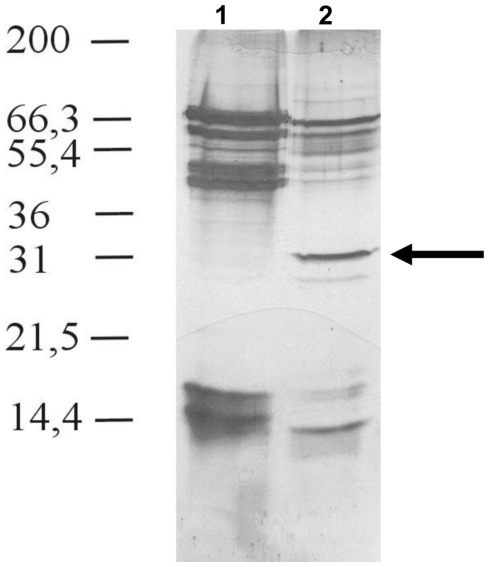
SDS-PAGE analysis of purified saliva from *C. nubeculosus and C. sonorensis*. Purified saliva proteins (20 µg) from *C. nubeculosus* (lane 1) and *C. sonorensis* (lane 2) were analysed on 15% SDS-PAGE and visualized using silver staining. A representative figure of at least four separate and independent saliva collections is shown. A cluster of saliva proteins was identified between 40–70 kDa and 13–17 kDa molecular weight for both species. However *C. sonorensis* saliva has an additional prominent protein band at around 29 kDa (arrow), which was identified as “late trypsin” using mass spectrometry analysis. The presence of this 29 kDa protein in *C. sonorensis* saliva was consistent in 5 independent saliva collections.

One dimensional electrophoresis identified a prominent ∼29–30 kDa protein in C. *sonorensis* saliva, however, comparing similar quantities of both species saliva the 29 kDa protein seemed to be absent from *C. nubeculosus* saliva (Figure 1). Similar protein profiles were obtained from at least 4 independent saliva collections and preparations from both species. One dimensional SDS-PAGE of large amounts (≥50 µg) of *C. nubeculosus* saliva proteins showed two faint bands in the area between 27–32 kDa, however the quantities of any proteins which migrated between 27 kDa–33 kDa were insufficient to allow identification by tandem mass spectrometry (not shown). Contrary the ∼29 kDa protein was one of the major identifiable proteins in at least 6 independently collected *C. sonorensis* saliva preparations. It was therefore concluded that the 29 kDa protein is present in *C. sonorensis* saliva at least in vastly greater amounts.

Two-D separation and mass spectrometry analysis of this protein from *C. sonorensis* saliva, generated the 17 amino-acid long sequence ‘TSAWSSSSDQLNFVDMR’. This is identical to a region of ‘late-trypsin’ previously reported from a *C. sonorensis* cDNA library (UniProt Q66UC8_9DIPT, total length 275 AA - [25]). The presence of late-trypsin in *C. sonorensis* saliva was confirmed by the presence of additional peptides in further mass spectrometry analyses (unpublished data). The predicted molecular mass of the whole protein is 29.5 kDa, which coincides with its position during 1-D SDS-PAGE (Figure 1). No visible differences were observed after these analyses, between the saliva proteins from adult *C. sonorensis* derived from insect colonies that had been bred to be susceptible, or refractory to BTV infection [28], [29] (data not shown).

A sequence comparison of late trypsin from *C. sonorensis*, using the NCBI protein comparison and UniProt database, showed that it is most closely related to other insect trypsin or serine proteases, including a trypsin from *C. nubeculosus* (67% identity), tryptase-2 from *Culex quinquefasciatus* (46% identity) and serine protease from *Aedes aegypti* (47% identity).

### Cleavage of the outer coat protein VP2 of different BTV strains by saliva from *C. sonorensis* (susceptible and refractory strains) and *C. nubeculosus*

Proteases such as chymotrypsin have previously shown to be able to cleave the outer-coat protein of purified BTV particles [30]. In order to determine if proteases identified in the saliva of *C. sonorensis* have a similar effect on the outer-coat protein VP2 of different BTV strains, purified virus particles were incubated at different temperatures in the presence and absence of saliva proteins. SDS-PAGE analyses of secreted saliva of *C. nubeculosus* [27], as well as those using whole salivary glands extracts [31] failed to identify detectable amounts of proteases similar to the late trypsin identified in *C. sonorensis* saliva. Additionally IAH Pirbright holds two colonies of *C. sonorensis*, which contain insect strains that were previously selected for their ability, or inability, to transmit BTV [29], [32], referred to as ‘susceptible’ and ‘refractory’ respectively. The ability of saliva proteins from both of the *C. sonorensis* strains and *C. nubeculosus* to cleave the outer coat protein of BTV was compared to identify any differences in the effects on BTV caused by saliva from vector or non-vector *Culicoides* species.

**Figure 2:**
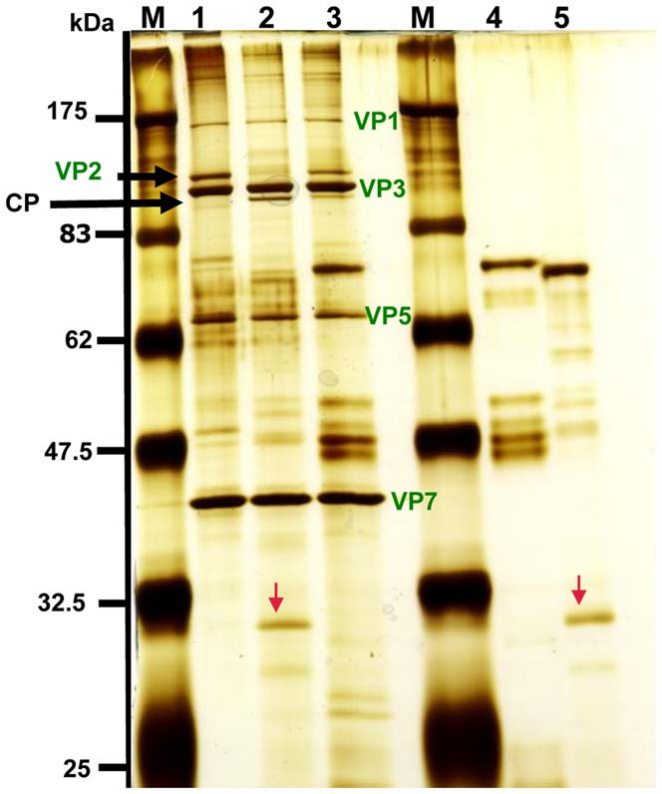
Saliva proteins from *C. sonorensis* cleave VP2 of BTV-16 more efficiently than saliva proteins from *C. nubeculosus*. Purified BTV-16 (RSAvvvv/16) virus particles (2 µg) were incubated for 3 hours at 37°C as a mock (lane 1) or with purified saliva proteins (2 µg) from either *C. sonorensis* or *C. nubeculosus* and BTV viral proteins were analyzed on a 12% SDS-PAGE using silver-staining for visualization. M represents the molecular weight markers. Lane 1: purified BTV-16 virus particles (2 µg); lane 2: purified BTV-16 virus particles incubated with *C. sonorensis* saliva proteins; lane 3: BTV-16 incubated with C. *nubeculosus* saliva proteins; lane 4: 2 µg of *C. nubeculosus* saliva proteins; lane 5: 2 µg of *C. sonorensis* saliva proteins. Outer capsid protein VP2 of BTV-16 (black arrow) was mostly cleaved by the *C. sonorensis* saliva proteins (lane 2), while only partial cleavage occurred with *C. nubeculosus* saliva proteins (lane 3). The cleavage of VP2 resulted in a cleavage product (CP) migrating at 110 kDa just underneath VP3. No further breakdown products of VP2 could be identified in these cleavage experiments. The red arrow indicates the 29 kDa trypsin like protein in *C. sonorensis* saliva and demonstrates its relative abundance compared to *C. nubeculosus* saliva. Figure 2 is a representative picture from 3 independent experiments.

Incubation of BTV-16 (RSAvvvv/16 or RSArrrr/16) virus particles with *C. sonorensis* saliva proteins resulted in complete cleavage of VP2 while only partial digestion of VP2 occurred after treatment with saliva proteins from *C. nubeculosus* under the same conditions (Figure 2, lanes 2 and 3). A large cleavage product (∼110 kDa) was generated (Figure 2).

**Figure 3:**
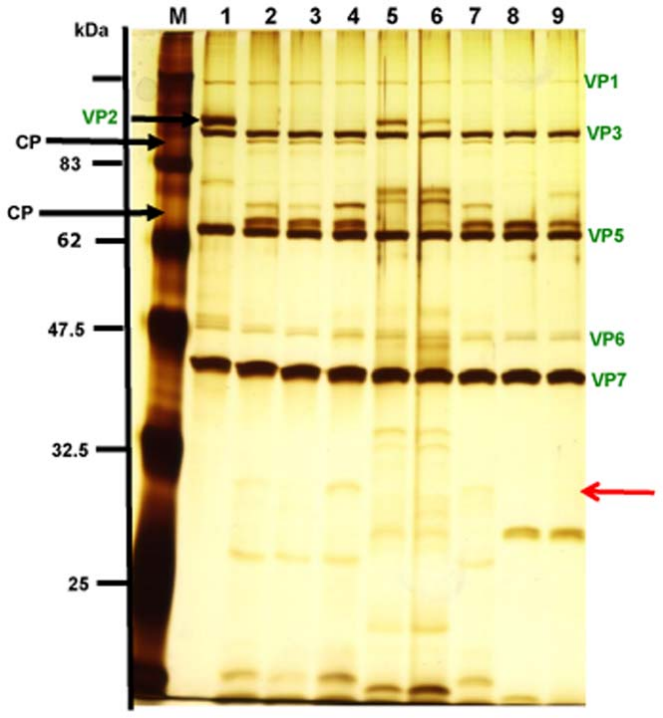
*C. sonorensis* saliva or trypsin control cleave VP2 of BTV-1 particles, more efficiently than *C. nubeculosus* saliva. Purified BTV-1 (RSArrrr/01) viral particles (2.5 µg) were incubated for 3 hours at 37°C either on its own as mock (lane 1) or with 0.5 or 1 µg saliva proteins from *C. sonorensis susceptible* (lanes 2 & 3), *C. nubeculosus* (lanes 5& 6) or *C. sonorensis refractory* (lanes 4&7) or with 1 or 0.5 µg trypsin as positive controls (lanes 8 & 9). M represents the molecular weight markers. Viral proteins were analysed by 10% SDS-PAGE and visualized by silver staining. BTV-1 VP2 protein (short arrow-lane 2) was completely cleaved by the saliva from both *C. sonorensis* strains (lanes 2&3 and 4&7) and by the control protease trypsin (lanes 8&9), resulting in two dominant cleavage products (CP) running at 110 kDa and 67 kDa respectively. Incubation with *C. nubeculosus* saliva proteins only resulted in partial cleavage of VP2 (lanes 5&6). VP2 cleavage products are either not detectable yet using 0.5 µg of *C. nubeculosus* saliva proteins (lane 5) or the smaller CP just starts to appear using 1 µg of *C. nubeculosus* saliva proteins. Due to a lower amount of saliva used many saliva proteins are not clearly identifiable on this SDS-PAGE. The 29 kDa sized protein identified as late trypsin (red arrow) is just visible in the lanes containing *C. sonorensis* saliva (lane 2,3,4 and 7).

Purified BTV-1 (RSArrrr/01) virus particles, incubated in the absence of proteases, contained the major structural proteins of BTV (Figure 3, lane 1). Treatment with 0.5 µg or 1 µg of *C. sonorensis* saliva proteins (lanes 1 and 2) resulted in complete cleavage of VP2, generating two major breakdown products migrating at ∼110 kDa and ∼67 kDa respectively. VP2 was also completely cleaved by treatment with either 1 or 0.5 µg of trypsin, generating the same breakdown products (Figure 3, lanes 8 and 9 ).

BTV-1 VP2 was completely cleaved by saliva proteins from both susceptible and refractory colonies of *C. sonorensis*, with similar efficiencies and cleavage products (Figure 3 lanes 2 and 3 vs. lanes 4 and7). Although similar sized breakdown products were generated, again only partial digestion of VP2 occurred after treatment with saliva proteins from *C. nubeculosus* under the same conditions (Figure 3 lanes 5 and 6).

**Figure 4:**
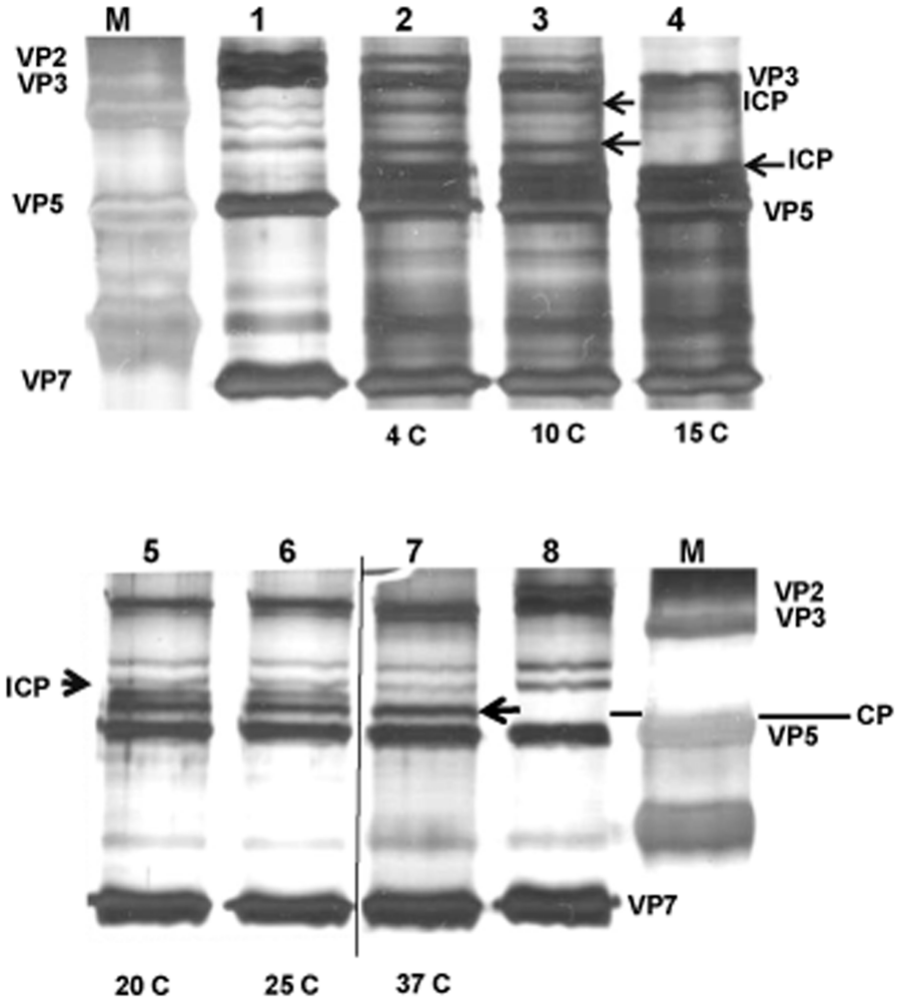
Efficiency of BTV-1 VP2 cleavage by *C. sonorensis* saliva is temperature dependent. BTV-1 particles (2 µg) were incubated with 1 µg of *C. sonorensis* saliva proteins at 4°C, 10°C, 15°C, 20°C, 25°C or 37°C for 2 hours in a final volume of 50 µl. Viral proteins were analysed on 10% SDS-PAGE and visualized using silver staining. Lane M shows molecular weight markers. Lane 1 and 8 are purified BTV-1 virus particles incubated at 37°C in the absence of proteases. After incubation at 4°C and 10°C (lane 2 and 3) intact BTV-1 VP2 was still visible, however some cleavage of VP2 had occurred resulting in intermediate cleavage products (ICP). At 15°C, no intact VP2 was visible but still only intermediate cleavage products (ICP) are generated. (lane 4). At higher temperatures, intermediate CP were completely cleaved to final CP (lane 7).

**Figure 5:**
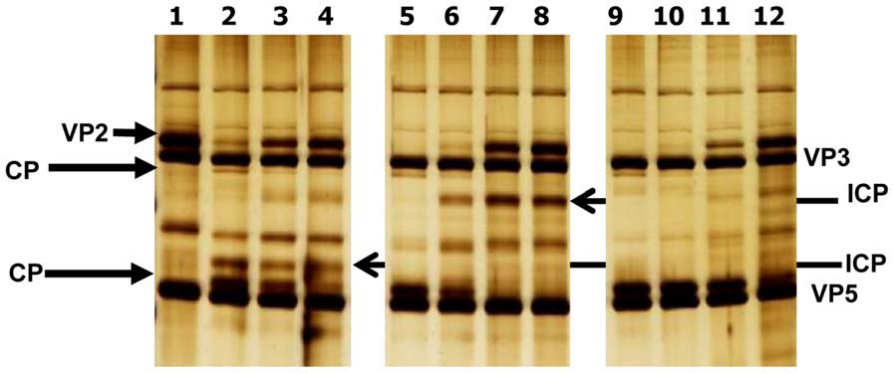
Comparison of the effect of different amounts of *C. sonorensis* saliva, trypsin and chymotrypsin. Purified BTV-1 virus particles (2.5 µg) were incubate at 37°C for 30 minutes in a final volume of 50 µl using different amounts of *C. sonorensis* saliva proteins (S) , trypsin (T) and chymotrypsin (CT). BTV proteins were analyzed on a 10% SDS-PAGE using silver-staining for visualization. Lane 1: no proteases; lane 2: +2 µg S; lane 3: +1 µg S; lane 4: +0.5 µg S; lane 5: +1 µg T; lane 6: +0.5 µg T; lane 7: +0.1 µg T; lane 8: +0.05 µg T; lane 9: +0.05 µg CT; lane 10: +0.01 µg CT: lane 11: +0.005 µg CT; lane 12: +0.001 µg CT. The same levels of BTV-1 VP2 cleavage were obtained with 2.0 µg of *C. sonorensis* saliva proteins, 0.5 µg of trypsin or an estimated 0.008 µg of chymotrypsin. For all three different proteases, *C. sonorensis* saliva, trypsin or chymotrypsin it can be seen that using lower amounts of proteases generates intermediate cleavage products (ICP) (e.g. lane 3 and 4, lane 7 and 8 and lane 12) which are further cleaved using higher amounts of proteases, leading to the two final VP2 cleavage products (CP) migrating at ∼110 kDa and ∼67 kDa respectively (e.g. lane 2, lane 5 and lane 9 and 10).

The efficiency of VP2 cleavage appeared to be a function of the amount of *C. sonorensis* saliva proteins in the reaction, incubation time and temperature. Although cleavage of BTV VP2 by *C. sonorensis* saliva proteins occurred over a wide range of temperatures, from 4°C–37°C, only partial cleavage of VP2 was detected under suboptimal conditions (Figure 4 and 5). Cleavage of VP2 BTV-1 (RSArrrr/01) was also shown to be a step-wise process, with intermediate cleavage products detected at lower temperatures (Figure 4) or lower amounts of proteases (Figure 5). At near optimal cleavage conditions these intermediate breakdown products are digested further until only the final breakdown products remain present (Figure 4 and 5).

### The susceptibility of EHDV-2 VP2 to cleavage by *Culicoides* spp. saliva differs between virus strains

In order to establish if the cleavage of the outer coat protein by *Culicoides spp.* saliva is a universal feature of orbiviruses, the effect of *C. sonorensis* saliva on two strains of EHDV-2 was investigated.

**Figure 6:**
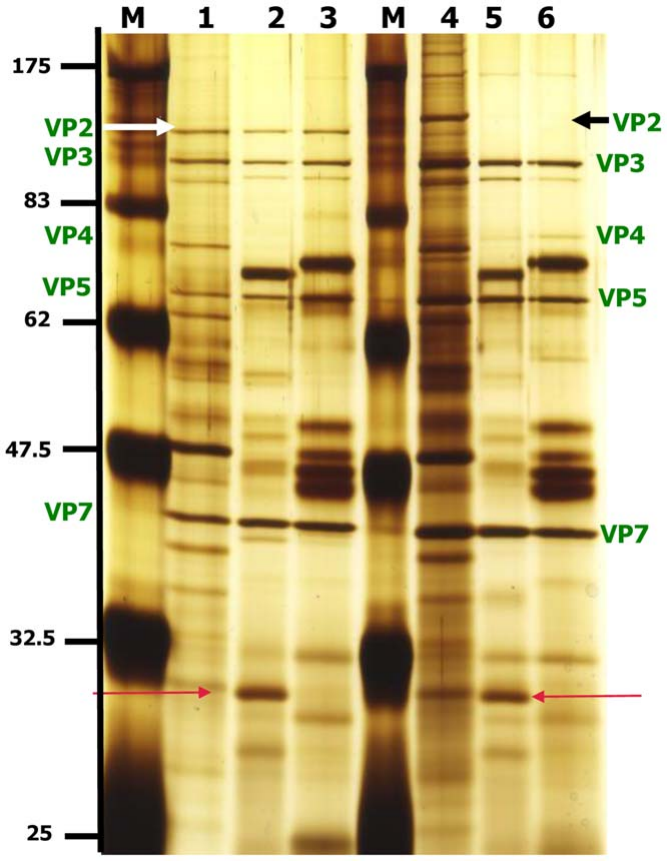
The ability of *Culicoides* saliva to cleave EHDV VP2 depends on the strain of EHDV. Purified EHDV-2 virus particles (1 µg) from two different strains, either CAN1962/01 or AUS1979/05, were incubated in a final volume of 50 µl for 3 hours at 37°C with or without saliva proteins (1 µg) from either *C. sonorensis* or *C. nubeculosus*. Viral proteins were analysed on 12% SDS-PAGE and visualised using silver staining. Lane M indicates molecular weight markers. Lane 1: CAN1962/01 virus particles; lane 2: CAN1962/01 virus particles with *C. sonorensis* saliva proteins; lane 3: CAN1962/01 virus particles with *C. nubeculosus* saliva proteins; lane 4: AUS1979/05 virus particles; lane 5: AUS1979/05 virus particles with *C. sonorensis* saliva proteins; lane 6: AUS1979/05 virus particles with *C. nubeculosus* saliva proteins. EHDV VP2 of the AUS1979/05 strain (black arrow) was cleaved by the saliva proteins from either *Culicoides* species and intact VP2 was not detected (lanes 5 & 6). In contrast EHDV VP2 of strain CAN1962/01 (white arrow) was still intact in the presence of saliva proteins from either *Culicoides* species (lanes 2 and 3). The black and white arrows indicate the VP2 proteins, which migrate at different size for the two different EHDV-2 strains. The red arrows highlight the 29 kD protein identified as late trypsin in the *C. sonorensis* saliva.

VP2 of an eastern EHDV-2 strain (AUS1979/05) [33] was completely cleaved by saliva proteins from either *C. sonorensis* or *C. nubeculosus* (Figure 6). In contrast, incubation of a western EHDV-2 strain (CAN1962/01) [33] with the saliva proteins from either *Culicoides spp.*, for 3 h at 37°C degrees, had little effect, and intact VP2 protein was still detected after incubation (Figure 6, lanes 2 and 3).

Although a band comparable to one of the major BTV-VP2 breakdown products (migrating slightly faster than VP3 at ∼110 kDa) was present in samples of AUS1979/05 after treatment with saliva, a band of a similar size was also present in the ‘mock-treated’ control lanes that also contained intact VP2 of both EHDV strains (Figure 6).

### Effect of protease inhibitors on the protease activity of *C. sonorensis* saliva ability to cleave BTV-1 VP2

In order to further investigate if the protease activity in *C. sonorensis* is solely due to the identified “late trypsin” and to compare the reactivity of the insect derived proteases and mammalian trypsin and chymotrypsin to inhibition, the cleavage of BTV-1 VP2 by these proteases was investigated in the presence or absence of different inhibitors.

**Figure 7:**
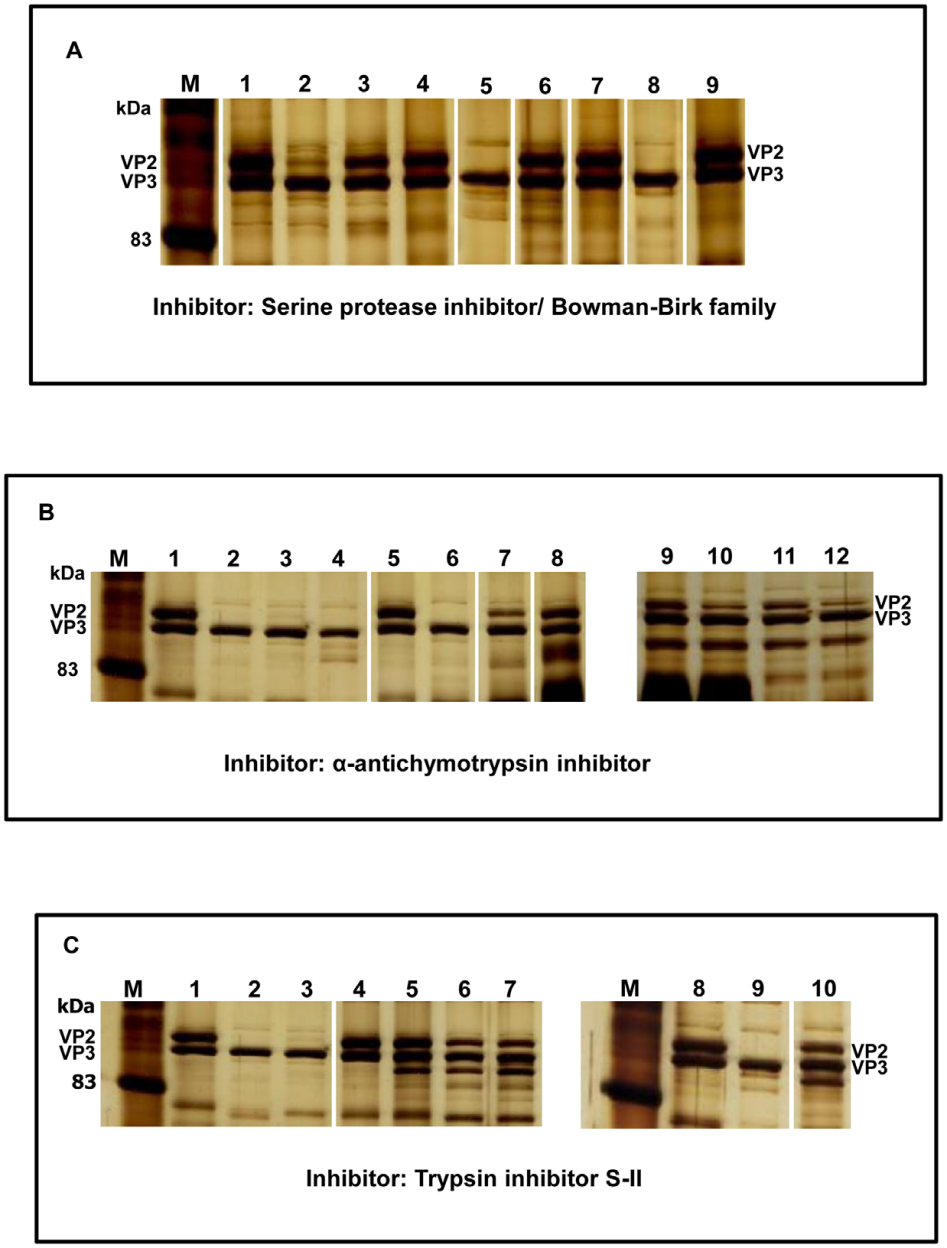
The effect of different protease inhibitors on *C. sonorensis* saliva, trypsin or chymotrypsin. BTV-1 purified virus particles were incubated with different amounts of *C. sonorensis* saliva proteins (S), trypsin (T) or chymotrypsin (CT) in a final volume of 50 µl for 30 minutes at 37°C, in the presence or absence of the following protease inhibitors : Bowman-Birk serine protease inhibitor (BBI); α-chymotrypsin inhibitor (CTInh) and Trypsin Inhibitor SII (Tinh). The viral proteins were analysed on 10% SDS-PAGE and visualized using silver staining. Lane M indicates molecular weight markers. Each reaction contained 2.5 µg BTV-1 particles with the different additions listed for each lane. Panel A: lane 1: mock treated virus particles, lane 2: +1 µg S, lane 3: +1 µg S and 1 µg BBI, lane 4: +1 µg S and 20 µg BBI, lane 5: +0.05 µg CT, lane 6: +0.05 µg CT and 1 µg BBI, lane 7: +0.05 µg CT and 20 µg BBI, lane 8: +0.5 µg T, lane 9: +0.5 µg T and 1 µg BBI. (Picture generated from one gel, not all lanes shown). Cleavage of BTV-1 VP2 by all three different proteases is prevented in the presence of the serine protease inhibitor, however *C. sonorensis* saliva proteins and chymotrypsin need a higher concentration of inhibitor to completely inhibit protease activity. Panel B: lane 1: mock treated virus particles, lane 2: +0.5 µg T, lane 3: +1 µg S, lane 4: +0.5 µg CT, lane 5: mock treated virus particles, lane 6: +0.5 µg T and 30 µg CTInh, lane 7: +1 µg S and 30 µg CTInh, lane 8: +0.5 µg CT and 30 µg CTInh, lane 9: +0.01 µg T and 30 µg CTInh, lane 10: +0.05 µg T and 30 µg CTInh, lane 11: +0.01 µg T; lane 12: +0.05 µg T. (Picture generated from two gels, not all lanes shown). The α-chymotrypsin inhibitor does not inhibit the cleavage of BTV-1 VP2 by trypsin but has a partial inhibition effect on the cleavage of VP2 by *C. sonorensis* saliva proteins. Panel C: lane 1: mock treated virus particles, lane 2: +0.5 µg T; lane 3: +1 µg S ; lane 4: mock treated virus particles, lane 5:+0.5 µg T and 2 µg TInh, lane 6: +1 µg S and 2 µg TInh, lane 7: +1 µg S and 4 µg TInh, lane 8: mock treated virus particles, lane 9: +0.05 µg CT, lane 10: +0.05 µg CT and 2 µg TInh (Picture generated from two gels not all lanes shown). Cleavage of BTV-1 VP2 by trypsin is completely inhibited by the trypsin inhibitor, however the protease activity of *C. sonorensis* saliva is only partially inhibited.

Cleavage of BTV-VP2 at any of the trypsin concentrations (0.05–0.5 µg/50 µl), or the lower concentrations of chymotrypsin (0.001 µg–0.01 µg/50 µl), was completely prevented by 1 µg Bowman-Birk serine protease inhibitor (BBI) ‘*Glycine max*’. Although VP2 cleavage by 1 µg *C. sonorensis* saliva proteins, or the higher concentrations of chymotrypsin (0.05 µg/ 50 µl) was only partially inhibited under these conditions, in both cases it was completely prevented by addition of 20 µg of BBI (Examples shown in Figure 7A).

The addition of 30 µg of α-antichymotrypsin to the reaction (50 µl) completely prevented cleavage of VP2 at any of the chymotrypsin concentrations used (0.001 µg–0.5 µg/ 50 µl) but had no affect on the cleavage of BTV-1 VP2 by trypsin (Examples shown in Figure 7B). Cleavage of BTV-1 VP2 by 1 µg *C. sonorensis* saliva proteins was partially inhibited by the addition of 30 µg of α-anti-chymotrypsin (Figure 7B).

Addition of 2 µg of trypsin-inhibitor S-II (TInh) to the reaction (50 µl) prevented cleavage of BTV-1 VP2 by 0.5 µg trypsin (Figure 7C), while cleavage by 0.5 µg chymotrypsin was unaffected (not shown). However, TInh (trypsin-inhibitor S-II) did have some effect on chymotrypsin, reducing cleavage by lower concentrations of the enzyme (0.05–0.001 µg/ 50 µl) (Figure 7C). Cleavage of BTV-1 VP2 by 1 µg *C. sonorensis* saliva proteins was also only partially prevented using 2 µg or 4 µg of the trypsin-inhibitor S-II (TInh) (Figure 7C).

### The effect of *C. sonorensis* saliva on BTV-1 infectivity for BHK and KC cells

It has previously been reported that infectious subviral particles (ISVP) of BTV generated by cleavage of VP2 using chymotrypsin, have a higher specific infectivity (10–100 times) for insect cells (KC cells) and adult *Culicoides*, as compared to the intact virus particle [23]. The possibility that that cleavage of VP2 by *C. sonorensis* saliva may have the same effect on the infectivity of the virus for insect cells was therefore investigated.

**Figure 8:**
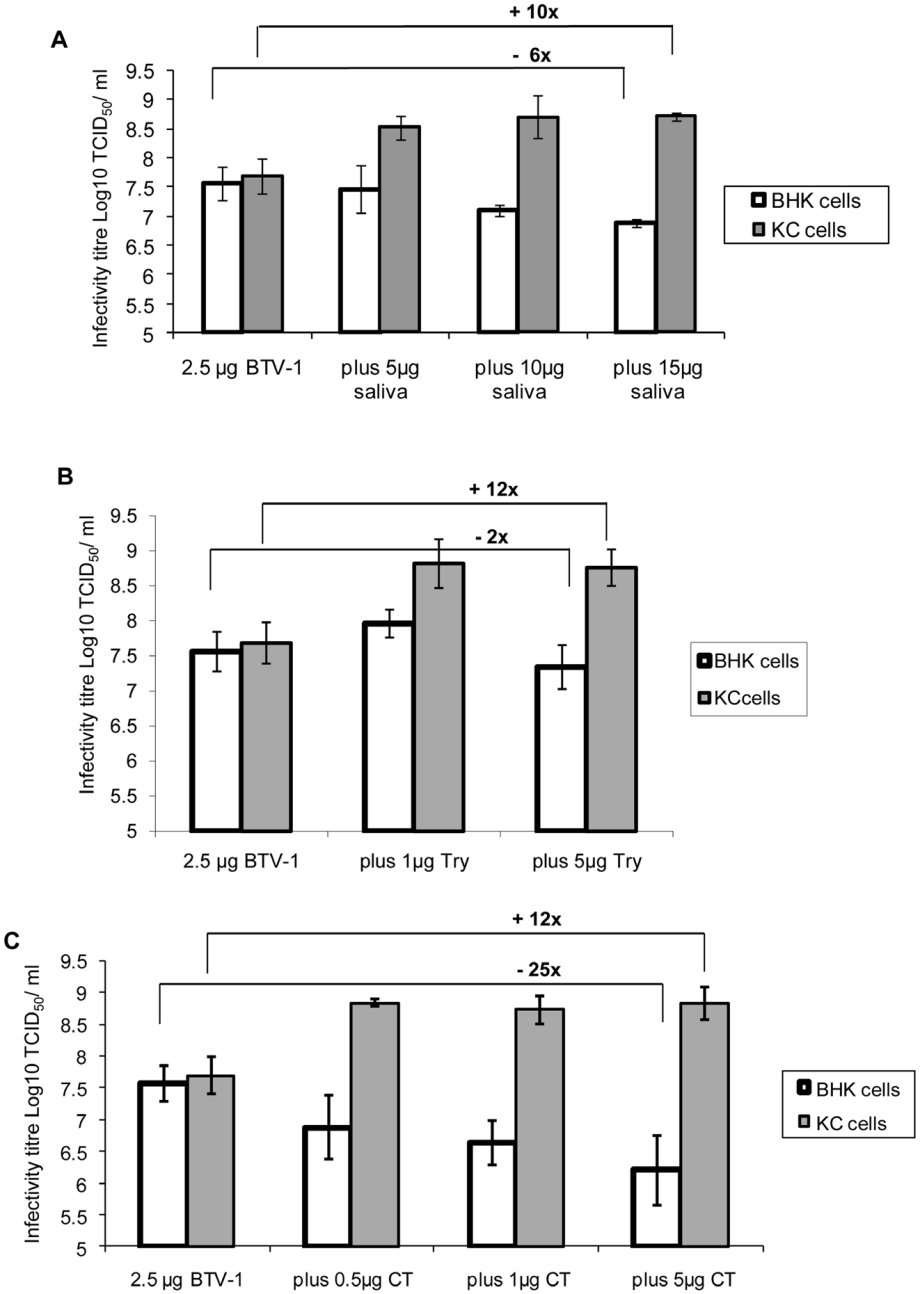
Infectivity of purified BTV-1 particles for BHK and KC cells following incubation with *C. sonorensis* saliva, trypsin or chymotrypsin. **Panel A**: BTV-1 purified particles (2.5 µg) were incubated either in the absence of proteases, or with 5, 10 or 15 µg of *C. sonorensis* saliva proteins, in a final volume of 100 µl for 3 hours at 37°C. **Panel B**: BTV-1 purified particles (2.5 µg) were incubated either in the absence of proteases or with 1 or 5 µg of trypsin (T), in a final volume of 100 µl for 3 hours at 37°C. **Panel C**: BTV-1 purified particles (2.5 µg) were incubated either in the absence of proteases or with 0.5, 1 or 5 µg of chymotrypsin (CT), in a final volume of 100 µl for 3 hours at 37°C. The treated BTV-1 particles were added to plain media (GMEM) and Log10 dilutions from −1 to −11 were prepared. These dilutions were titrated in either BHK cells or KC cells in 96 well plates (50 µl/ well and 8 wells/ dilution) and incubated at 37°C or 30°C respectively. The BHK cells were examined for CPE on day 5 and 7 post inoculation. The KC cells were ‘back titrated’ onto new BHK cell plates 7 d.p.i. and the titre determined by reading CPE on day 5 and 7 post transfer. Each data point represents at least 3–6 individual experiments (standard deviation = error bar). Cleavage of BTV-1 VP2 by different proteases increased the infectivity of modified particles for KC cells by 10 to 12 fold, while it reduced the infectivity for BHK cells between 2 and >20 fold.

Treatment of purified virus particles of BTV-1 (2.5 µg) with higher concentrations of *C. sonorensis* saliva proteins (10 or 15 µg per 100 µl reaction), increased their specific infectivity for KC cells by ∼10 fold (from an average of 10^7.7^ TCID_50_/ml to an average of 10^8.7^ TCID_50_/ml - Figure 8A) but reduced their infectivity for BHK cells by ∼3–6 fold (from an average of 10^7.6^ TCID_50_/ml to an average of 10^6.8^ to 10^7.1^ TCID50/ml). Addition of 5 µg of the *C. sonorensis* saliva proteins produced a similar but less pronounced effect, with the virus titre on KC cells being increased to an average of 10^8.5^ TCID_50_/ml, however, the titre for BHK cells remained almost unchanged (at 10^7.6^ TCID_50_/ml).

Treatment with either 1 or 5 µg of trypsin also increased the infectivity of BTV-1 for KC cells by ∼12 fold (to an average of 10^8.8^ TCID_50_/ml - Figure 8B). A 2 fold increase in infectivity for BHK cells was observed after treatment with 1 µg of trypsin, while a 2 fold decrease was detected using 5 µg of trypsin (Figure 8B).

Three different amounts of chymotrypsin (0.5 µg, 1 µg and 5 µg per reaction) were used to treat purified particles of BTV-1. In each case an increase in specific infectivity of least 10 fold was observed for KC cells (from an average of 10^7.7^ TCID_50_/ml to ∼10^8.8^ TCID_50_/ml). In contrast the infectivity of treated particles for BHK cells decreased by 10 to 25 fold (from an average of 10^7.6^ TCID_50_/ml to 10^6.2^ TCID_50_/ml for 5 µg of chymotrypsin respectively) (Figure 8C).

## Discussion

The saliva of adult *Culicoides* contains proteases that can cleave VP2 of BTV and at least some strains of EHDV. This cleavage changes the structure of the virus particle outer capsid, generating infectious subviral particles (ISVP), and thereby increases the infectivity of treated BTV particles ∼tenfold for KC cells (derived from *C. sonorensis*) (Figure 8). This represents the first demonstration that the structure and infectivity of a pathogen can be directly modified by saliva proteases from its insect vector.

Purification methods previously developed for different BTV particle types [30] had shown that VP2 in the BTV outer capsid can be cleaved by chymotrypsin, and that generated ISVP have an enhanced infectivity for KC cells and adult *Culicoides* [23].

The saliva of *C. sonorensis*, a major North American vector for BTV, cleave VP2 more efficiently than those from *C. nubeculosus*, which is a ‘poor’ or ‘non-vector’ species [34]. Electrophoresis and mass spectrometry showed that ‘late trypsin’ (∼29 kDa), identified through sequence homology with a ‘late trypsin’ previously reported in a cDNA expression library of *C. sonorensis* salivary glands (AY603563/ Q66UC8_9DIPT - [25]), is a relatively abundant component of *C. sonorensis* secreted saliva (Figure 1).

Although *C. nubeculosus* saliva also showed some protease activity, it contained only a very ‘faint’ protein band at ∼29 kDa during SDS-PAGE, which provided insufficient material for protein sequencing.

A cDNA expression library established from salivary glands of *C. nubeculosus* by Russell et. al. (2009) [26], reported two trypsin like proteases. These proteins, which had predicted sizes of 27.8 kDa and 35.03 kDa (accession number ACM4087 and ACM40901 respectively), are distinctly different in size and sequence from the late trypsin identified from *C. sonorensis* (AY603563- [25]). The saliva protein sequences that were generated from cDNA libraries for *C. sonorensis* [25] or *C. nubeculosus* [26] also show only relatively low levels of sequence homology, suggesting that homologous proteins are not highly conserved between these two species [26], [31]. Additional SDS-PAGE analyses of secreted saliva proteins of *C. nubeculosus* [27], as well as those using whole salivary glands extracts [31] also failed to detect any obvious proteins between 25 kDa and 37 kDa, despite the use of both 1 and 2-D gel analysis and/ or direct sequencing by mass spectrometry. These data indicate that trypsin like proteases are present in much lower quantities in *C. nubeculosus* saliva. These differences in the protein sequence, composition and relative quantities of individual protease(s) in insect vector saliva, potentially could modulate the vector competence levels of different *Culicoides* species.

Certain ‘barriers’ have been shown to exist within individual adult *Culicoides*, which can prevent or constrain the initial infection, replication and internal dissemination of ingested orbiviruses. They include: a mesenteron infection barrier (MIB); a mesenteron escape barrier (MEB); and a dissemination barrier (DB) [24], [28], [35].

Differences between the saliva proteins (particularly the proteases) between vector and non-vector *Culicoides spp.* and their relative ability to digest VP2, enhancing the infectivity of ingested orbivirus particles, could affect the initial stages of virus/cell interactions and infection of the insect midgut. These differences could therefore represent an important component of the MIB. Previously it was demonstrated that proteolytic enzymes, including those present in the mosquito midgut, can remove the outer coat glycoprotein G1:a of La Crosse virus (*Bunyaviridae*), thereby increasing virus affinity for mosquito cells [36]. The *Culicoides sonorensis* midgut also contains proteases, as demonstrated in a cDNA-library expression study [25] and the infectivity of a BTV-1 strain for adult *C. sonorensis* was reduced if the virus–bloodmeal contained protease inhibitors [23]. However the efficiency of BTV VP2 cleavage by *Culicoides* midgut proteases, and whether these midgut proteases vary in cleavage efficiency between *Culicoides* species, is currently unknown.

The work presented here not only shows that saliva proteases in the saliva of the competent vector *C. sonorensis* can effectively cleave BTV VP2 to form ISVP, it also demonstrates the cleavage efficiency of BTV VP2 is influenced by the enzyme-virus particle ratio. Ingestion of the insect's saliva will increase the overall level of proteases present in the gut environment, thereby also increasing the relative efficiency of particle conversion to ISVP (and consequently infectivity for the insect). Uptake of *Culicoides* saliva by the feeding insect is very likely due to the nature of the ingestion mechanism. Unlike mosquitoes *Culicoides* are ‘pool’ feeders [37]; using a rasping proboscis to create a small wound in the skin, followed by the uptake of the material influx into this “pool” – which includes blood, lymph, lymph cells and the inoculated saliva. Therefore it is likely that during this process at least part of the BTV outer-coat cleavage by saliva enzymes takes place within the mammalian host's skin, which is held at 30–37°C. This not only gives a period for interaction between virus and saliva proteases prior to ingestion, it also makes this stage of the digestion mechanism more independent of environmental temperatures.

The saliva proteins collected and purified from vector competent and refractory colonies of *C. sonorensis*, were very similar by SDS-PAGE, and there was no detectable differences in their efficiency of VP2 cleavage. However as only a proportion of individuals within the competent vector colony of *C. sonorensis* will become infected, further work is needed to investigate saliva protein compositions between competent and refractory individuals.

Cleavage of VP2 from BTV-1 or BTV-16 (western and eastern BTV topotypes respectively – [38]) by *Culicoides* saliva proteins, suggests that this may be a generalised mechanism involved in the uptake and / or transmission pathway of different BTV serotypes and topotypes. Although VP2 of an ‘eastern’ EHDV-2 ‘AUS1979/05’ was also cleaved by treatment, VP2 of the western EHDV strain ‘CAN1962/01’ remained intact, despite prolonged incubation. This indicates that not all orbiviruses have VP2 cleavage sites that are appropriate for the saliva proteases of these particular *Culicoides* species. VP2 from both of these EHDV-2 strains has a predicted size of 982 AA, with 26.2% amino acid sequence variation between them, although the major VP2 band of AUS1979/05 migrates slightly more slowly during SDS-PAGE [33], [39] (Figure 6). Numerous potential trypsin and / or chymotrypsin cleavages sites were identified during sequence analysis of Seg-2 / VP2 from CAN1962/01 or AUS1979/05, some of which are unique to either protein. If one or more of these unique sites is essential for VP2 cleavage it may explain the stability of the protein from CAN1962/01.

The protease activity of *C. sonorensis* saliva appears likely to be mediated by one or more serine proteases, as indicated by its sensitivity to the Bowman-Birk inhibitor, and is likely to be at least partly due to the late trypsin identified here. However, the protease activity of *C. sonorensis* saliva seemed to be less sensitive to inhibition by BBI or trypsin inhibitor (S-II) than mammalian trypsin or chymotrypsin (Figure 7A and 7C). *C. sonorensis* saliva proteases were also partially sensitive to inhibition by α-antichymotrypsin. This suggests that a second (chymotrypsin like) serine protease may also be present in the saliva, which is inhibited by α-antichymotrypsin, allowing partial cleavage of BTV-1 VP2 by the unaffected trypsin activity (Figure 7B). However no other serine protease (apart from the late-trypsin) was identified during 2-D gel electrophoresis or mass spectrometry of *C. sonorensis* saliva, or by salivary glands transcriptome analysis [25]. Alternatively, given the structural differences between trypsin like proteases derived from insects and mammals it could be possible that the *Culicoides* derived trypsin- like proteases react less sensitive or less specific with these inhibitors than their mammalian counterpart.

Protease treatment of purified BTV-1 virus particles can significantly increase their specific-infectivity for KC cells (Figure 8). This increase in BTV-1 infectivity was more pronounced at the higher proteases concentrations used (Figure 8) and initial results (not shown) showed minor changes in infectivity using relatively low amounts of proteases (e.g. 0.1 µg chymotrypsin, 0.5 µg trypsin or 1 µg *C. sonorensis* saliva proteins), despite the fact that BTV-1 VP2 was almost completely cleaved by those smaller amounts of protease when tested by SDS-PAGE (Figure 5). The cleavage of BTV-1 VP2 at different temperatures or different concentrations of *C. sonorensis* saliva proteins suggest that intermediate breakdown products are initially formed, which are then processed further (Figures 4 and 5). It therefore appears likely that the initial cleavage of VP2 does not in itself alter BTV infectivity, and further processing or cleavage of VP2 is needed to increase specific infectivity.

The importance of saliva-modified virus-particles during transmission of BTV from the insect to the ruminant host is currently unknown. Previous analyses of *C. nubeculosus* or *C. sonorensis* have identified protease inhibitors in the saliva [25], [27]. These may at least partially inhibit the proteolytic enzymes within the salivary gland, only allowing them to become fully active once the inhibitors are diluted and the temperature is increased during feeding on the mammalian host. However, it appears likely that these proteolytic enzymes will modify the virus particles that are generated by replication within the salivary glands, during or immediately after their inoculation. As a consequence ISVP or core particles could play a significant role in the early stage of infection of the mammalian host.

In the work presented here, treatment of purified BTV particles with *Culicoides* saliva proteins, modified their outer-capsid proteins and reduced overall infectivity for BHK cells. In contrast, previous studies showed that when crude cell culture material was treated with proteases prior to purification these purified BTV ISVP retained a similar specific-infectivity to that of the fully intact virus particle for BHK cells and, similar to results presented here, an increased infectivity for KC cells [23], [30]. Additionally purified core particles were much less infectious for BHK cells [23] and were entirely non-infectious for CHO cells (Mertens unpublished), but retained a high level of infectivity for KC cells. The studies reported here, treated already purified and intact virus-particles with proteases, and did not remove the enzymes after treatment. This could potentially lead to further processing of at least some of the particles to cores after the initial cleavage events, possibly explaining the reduced infectivity for BHK cells observed.

In addition, although KC cells have so far shown to be a good *in vitro* representation for *C. sonorensis* (from which larvae these cells have been generated) [23], [40], BHK cells, a standard cell line commonly used for BTV *in vitro* studies, are not suited to represent all of the so far identified numerous mammalian cell targets of BTV.

Within its mammalian hosts BTV is reported to infect a number of very different cell types, such as endothelial cells, monocytes, dendritic cells and lymphocytes [41], [42], [43] and all of these cells may exhibit different susceptibilities to the intact virus particles, ISVP or cores generated during interaction with the insect saliva. Previous studies have already shown that ISVP have lost their haemaglutination activity, indicating that their cell binding properties have changed, particularly for red blood cells. Additionally leukocytes are known to express a number of integrin receptors [44] some of which can bind RGD motifs, similar to those which are present on the upper outer surface of the BTV VP7 trimers on the core-particle surface [45], [46]. It has also previously been demonstrated that feeding of *C. sonorensis* on sheep skin induces a chemo-taxis' effect, recruiting inflammatory leukocytes to the biting side, thereby providing potential cellular targets for BTV infection [47], [48]. Future work will therefore investigate if *Culicoides* saliva also plays an important role for the infectivity and virulence of BTV particles for some of the cellular targets within its natural mammalian host.

In summary, the data presented in this paper demonstrated that proteases in the saliva of adult *Culicoides* can directly modify the structure and the infectivity of BTV and possibly other orbiviruses they transmit, through cleavage of the outermost viral protein VP2. This indicates that the transmission efficiency of BTV and related orbiviruses is likely to be influenced by the composition and protease activity of the insect vectors' saliva, as well as ambient temperatures, the protease sensitivity of the virus outer coat protein, and the host cell susceptibility to protease cleaved particles (both in the vector and ruminant).

The findings presented in this paper may not only be of relevance for orbiviruses and their *Culicoides* vector. The possibility remains that other arboviruses could also be modified directly by their arthropod vector saliva, and to elucidate such mechanisms would be of major importance to fully understand and eventually prevent arbovirus transmission.

## Materials and Methods

### *Culicoides spp.*

Colonies of two different *Culicoides* species are maintained in the insectary at the Pirbright Laboratory, of the Institute for Animal Health. The colony of *C. nubeculosus* (a European species considered to be a poor or “non-vector” species for BTV) has been maintained for more than 30 years. The colony of *C. sonorensis* (considered to be the major BTV vector in North America) was originally established at the USDA laboratory in Laramie, and an ‘offshoot’ colony has been maintained at IAH Pirbright for >30 years [49]. The *C. sonorensis* colony includes two distinct strains, one of which has been selected to be ‘vector-competent’ for orbivirus transmission (e.g. BTV and AHSV), while the other ‘refractory’ colony was selected for low vector competence for AHSV and BTV [29], [32].

### Collection and purification of *Culicoides* saliva proteins

The collection and purification of *Culicoides* saliva proteins was carried out as described previously for *C. nubeculosus* [27]. Briefly saliva was collected by modifying an artificial midge feeding systems developed for *C. nubeculosus* and *C. sonorensis* [50]. The feeding system consists of an inner and an outer glass chamber. The inner chamber was either filled with heparinised horse blood or a sugar solution and sealed with a Parafilm™ (American National Can, Greenwich, United States). The outer chamber was connected to a water circulation system to maintain the blood at 37°C. For collection of saliva, a Durapore™ filter membrane (Millipore, Eschborn) was soaked in sucrose solution (5% w/v - to encourage midge feeding) and placed on top of the Parafilm™. Finally another layer of Parafilm™ was placed above the filter membrane to prevent contamination with non-salivary proteins from the midges. Pill boxes, containing 250–300 adult *Culicoides* (both sexes) of either *C. nubeculosus* or *C. sonorensis* were then attached to the saliva collection system for 20 to 30 min. Saliva proteins were eluted from the filter membranes in 1 mM CHAPS (OMNILAB) in 50 ml buffer (either PBS or 0.2 M Tris) on a shaker, overnight at 4°C. The eluted proteins were washed and concentrated to a final volume of 500–1000 µl by ultra-filtration using VIVASPIN falcon tubes (size cut-off 3 kDA, Vivascience, Hanover, Germany).

The method was developed using *C. nubeculosus* and proteins recovered were sequenced by mass spectrometry as described [27]. All proteins identified were associated with insect saliva; therefore the method was regarded as successfully generating saliva proteins in the absence of contaminating proteins from other sources.

Here *C. sonorensis* saliva proteins to be used in cell cultures were passed through 0.2 µm filters and subsequently concentrated via sterile ultra-filtration using VIVASPIN falcon tubes, then washed with a three-fold volume of sterile 0.2 M Tris/HCl (pH 8).

As the collected *Culicoides* saliva was washed and concentrated and all quantification were based on protein quantities, the end-product of the saliva collection is termed “saliva proteins” throughout this manuscript.

### Protein quantification

Quantification of saliva proteins was performed using the BCA™ Protein Assay (Pierce Prod# 23227) as described by the kit manufacturer. The optical density was read in a DYNEX MRX ELISA reader and the protein concentrations calculated in relation to standardized samples, using the DYNEX REVALATION program.

### Mass spectrometry and identification of late trypsin in *C. sonorensis* saliva proteins

The saliva proteins of *C. sonorensis* were analysed by electrophoresis and mass spectrometry as described for *C. nubeculosus* saliva proteins [27]. Briefly, the individual saliva proteins were separated by 2-D SDS-PAGE and the gel was stained with silver, as described below. The protein spot, which represented a major difference between *C. sonorensis* and *C. nubeculosus* (later identified as trypsin) was excised and analysed by mass spectrometry using the Electrospray Ionization Quadruple Time of Flight (QToF)-Tandem Mass Spectrometry. Amino acid sequences were used to search the UniProt and/or NCBI database using MASCOT or the FASTA program.

### SDS-Page analysis and silver staining

Two volumes of protein solution were added to one volume of 3× sample buffer (3× SDS sample buffer, substituted with 10% DTT at 1.25 M; New England Biolabs) and heated for 5 min at 100°C. SDS-PAGE was carried out using 10%, 12% or 15% polyacrylamide resolving gels, with a 5% stacking gel as described [51]. Electrophoresis was carried out either using the Protean II electrophoresis set (Bio-Rad) using 1.5 mm thick gels or the MINI PROTEAN II cell system (Bio-Rad) with 1 mm thick gels at 240 V/40 mA or 200 V/400 mA respectively. Proteins were fixed in the SDS-PAGE using 30%methanol+10% acetic acid in dH_2_O.

Gels were silver stained using the SilverSnap® kit (Pierce) according to the manufacturer's instruction. Pre-stained protein electrophoresis markers (New England Bio Labs) was used to estimate the molecular masses of individual saliva proteins.

### Virus and virus purification

The viruses used in this study were three strains of BTV: namely BTV-1 (RSArrrr/01), the reference and the vaccine strains of BTV-16 (RSArrrr/16 and RSAvvvv/16): and two strains of EHDV-2 (an ‘eastern’ strain AUS1979/05 and a ‘western’ strain CAN1962/01), all of which were obtained from the IAH reference collection (http://www.reoviridae.org/dsRNA_virus_proteins/ReoID/BTV-isolates.htm). All viruses used were purified as described before [30]. Briefly 40 roller bottles containing BHK cells at 90% confluence were infected with the appropriate BTV or EHDV strain. When 100% CPE was achieved virus particles were purified using discontinuous sucrose density gradients.

### Virus particle - saliva protein incubation

Incubation of purified orbivirus particles with proteases was carried out in a 50 µl volume for SDS-PAGE analysis, while 100 µl reactions were used for cell titrations to measure particle infectivity. BTV-1 particles were used at a concentration of 2.5 µg (12.5 µl) per reaction. Enzymes were added (as indicated) and the final volume was made up by adding 0.2 M TRIS/HCl, pH 8. The samples were incubated in an Eppendorf PCR machine, to guarantee constant temperatures, at temperatures (usually at 37°C) and time periods specified in individual experiments. The same batches of *C. sonorensis* saliva, trypsin (Sigma T4549) or chymotrypsin (Sigma C3142) were used for all assays investigating enzyme activity. The aliquots of the trypsin and the *C. sonorensis* saliva were kept at +4°C for 6 weeks without losing activity, but as chymotrypsin was unstable at +4°C its aliquots were kept at −80°C.

### Protease inhibitor and BTV VP2 cleavage

Purified BTV-1 particles (RSArrrr/01) were incubated with different amounts of trypsin, chymotrypsin and *C. sonorensis* saliva proteins, either with or without the respective protease inhibitors and the final volume (50 µl) was made up by adding 0.2 M TRIS/HCl, pH 8. Protease inhibitor used for these studies were a serine-protease inhibitor from the Bowman-Birk inhibitor family (Sigma, T9777), α-antichymotrypsin (Sigma A9285) and trypsin-inhibitor S-II (Sigma, T9128).

### Cell cultures

BHK-21 cells (European Collection of Cell Cultures (ECACC)) were grown in GMEM medium, containing 5% adult bovine serum (ABS), 5% tryptose phosphate broth (TPB) and antibiotics (100 U penicillin/ 10 µg streptomycin per ml) at 37°C with 5% C0_2_. KC cells [40], an embryonic cell line established from *Culicoides sonorensis* larvae [40] were grown in Schneider's insect medium supplemented with 10% FCS, antibiotics (100 U penicillin/ 10 µg streptomycin/ 2.5 µg Amphotericin B per ml) at 26–30°C.

### Titration on insect and mammalian cell lines

BHK cells (approximately 5×10^5^ in 100 µl growth medium) were added to every well of a 96-flatwell micro-titre plate and incubated over night at 37°C with 5% CO_2,_ to achieve 80% confluence. The growth medium was replaced with 150 µl maintenance medium, prior to use for virus titration. KC cells in 96 well plates were prepared immediately before the titration with the addition of a cell-suspension (approximately 1×10^6^ KC cells in 150 µl Schneider's growth medium) to each well.

Hundred µl of virus particles (either intact or incubates with saliva/ proteolytic enzymes) were added to 900 µl of GMEM medium and a ten-fold dilution series up to 10^−11^ was prepared. Fifty µl of the appropriate dilution was added to each well (as 8 fold duplicates/plate) of 96-well plates containing BHK or KC cells. Media was added to one row as a negative control. The CPE developed in BHK cells was read at 5 and 7 days post inoculation and titres were calculated as described by Kaerber (1931) [52]. As KC cells do not show CPE, 50 µl of supernatant from each well was transferred to a newly prepared BHK cell plate at 7 days post inoculation., creating an exact mirror of the original KC cell plate as a ‘back titration’. The viral titre shown by the new BHK cell plate was determined at 5 and 7 days post transfer. The BHK cell plates (1^st^ round of titration) were also transferred to newly prepared BHK cells to examine if the virus titre would change, however, as no changes in viral titre were observed, this practice was discontinued.
